# Degeneration and energy shortage in the suprachiasmatic nucleus underlies the circadian rhythm disturbance in ApoE^−/−^ mice: implications for Alzheimer’s disease

**DOI:** 10.1038/srep36335

**Published:** 2016-11-08

**Authors:** Lan Zhou, Qian Gao, Meng Nie, Jing-Li Gu, Wei Hao, Lin Wang, Ji-Min Cao

**Affiliations:** 1 Department of Physiology, Peking Union Medical College, Chinese Academy of Medical Sciences, Institute of Basic Medical Sciences, 5 Dong Dan San Tiao, Beijing 100005, China

## Abstract

Alzheimer’s disease (AD) patients suffer sleep disorders and circadian rhythm disturbances (CRDs). The underlying mechanisms are incompletely understood, and treatments are lacking. In this study, we characterized the locomotor activity, clock gene expression, morphological degeneration and energy metabolism of suprachiasmatic nucleus (SCN), together with retinal light sensing, in ApoE^−/−^ mice, a model for AD. Compared with the control C57BL/6J mice, ApoE^−/−^ mice exhibited disordered circadian locomotor activity under dim light and constant darkness, with impaired re-entrainment to phase change schedules. Decreased retinal melanopsin expression, together with amyloidosis and tau deposition, was evident in ApoE^−/−^ mice. Mitochondrial and synaptic deterioration, altered SIRT1-mediated energy metabolism and clock gene expression were also observed in ApoE^−/−^ SCN. Supplementation with fat or ketone bodies but not glucose, or intraperitoneal administration of nicotinamide, restored the locomotor rhythmicity and circadian expression of SIRT1 and clock genes, as well as reducing neurodegeneration. Taken together, ApoE deficiency induced degeneration and a significant disturbance in the SCN rhythmicity. Decline of retinal light sensing and SCN structural and metabolic deteriorations represented the major pathologies accounting for the CRDs in ApoE^−/−^ mice. Our curative experiments may help develop future therapies to treat the CRDs and sleep disorders in AD patients.

Sleep disturbances are widespread among the elderly. Degenerative neurologic disorders that cause dementia, such as Alzheimer’s disease (AD), exacerbate age-related changes in sleep. Many AD patients suffer multiple sleep disorders^1^,^2^. Common symptoms include nighttime sleep fragmentation, increased sleep latency, decreased slow-wave sleep, and increased daytime napping^3^,^4^. “Sundowning”, a phenomenon common to AD patients and elderly, is believed to be related to circadian rhythm disturbances (CRDs) that cause psychological and behavioral changes such as psychomotor agitation worsened specially during the late afternoon or early evening^5^. Furthermore, mounting evidence suggests the CRDs in AD are not only a consequence of amyloidosis and cognitive decline, but may also trigger or affect the disease progression^4^,^6^.

In mammals, the circadian rhythm of many physiological and behavioral processes including body temperature and sleep-wake cycle is coordinated by a suprachiasmatic nucleus (SCN) central clock^7^. The SCN is highly sensitive to retinal light signals. Environmental light can reset the central clock so as to adjust the endogenous activity rhythms to adapt to external illuminative conditions, a process termed light entrainment. In this process, melanopsin, which is mainly expressed in the retinal ganglion cells (RGCs), senses the environmental light as a photoreceptor and transduces the light signal to the SCN through the retinohypothalamic tract. In this sense, the melanopsin level and light sensing activity affects the SCN central clock function. Recent studies suggest that the SCN central clock is also affected by internal metabolite levels including blood glucose and lipids^8^,^9^.

Apolipoprotein E (ApoE), an essential lipid transport protein, is a major genetic risk factor for AD^10^. For example, ApoE ε4 allele has been recognized as a causative genetic factor for AD. In the central nervous system, ApoE mediates the mobilization and redistribution of cholesterol and phospholipid in membrane remodeling associated with synaptic plasticity. ApoE deficient (ApoE^−/−^) mice represent a model for AD induced by metabolic dysfunction in addition to amyloidosis. The clock and atherosclerosis-related genes were found differentially expressed in ApoE^−/−^ mice. For example, ApoE^−/−^ mice showed phase delay in the circadian pattern of serum lipid levels^11^, altered expression of clock^12^ and atherosclerosis/thrombosis-related genes^13^.

Sleep disruption is one of the major clinical manifestations in AD patients, but the underlying mechanisms are incompletely understood. Circadian clock cooperates with the forebrain to generate the sleep-wake cycles. A transgenic model of AD expressing the three genetic mutations of amyloid precursor protein (APP), Presenilin-1 and tau associated with familial AD (APP_Swe_, PS1_M146V_ and tau_P301L_) showed decreased nocturnal activity and increased daytime activity, with shorter free-running periods^14^. These results suggest that neurodegeneration such as amyloidosis disrupt normal circadian rhythmic activity. Vasoactive intestinal polypeptide (VIP)- and vasopressin-containing neurons were reduced in the SCN of these transgenic mice^14^. Other transgenic AD mouse models expressing single APP mutation also showed changes in core body temperature rhythm and locomotor activity, but the CRD severity and neurodegeneration extent seemed to depend on the particular nature and multiplicity of APP mutations^15^,^16^,^17^,^18^.

To the best of our knowledge, the circadian rhythmicity, especially the light entrainment of locomotor activities, has not been characterized in ApoE^−/−^ mice. The main purpose of this study was to characterize the CRDs in ApoE^−/−^ mice and unravel the underlying mechanisms, with a focus on SCN and RGC. Based on our findings, we also devised novel dietary and pharmacological interventional regimens to restore the normal circadian rhythmicity and reduce neurodegeneration.

## Results

**Figure 1:**
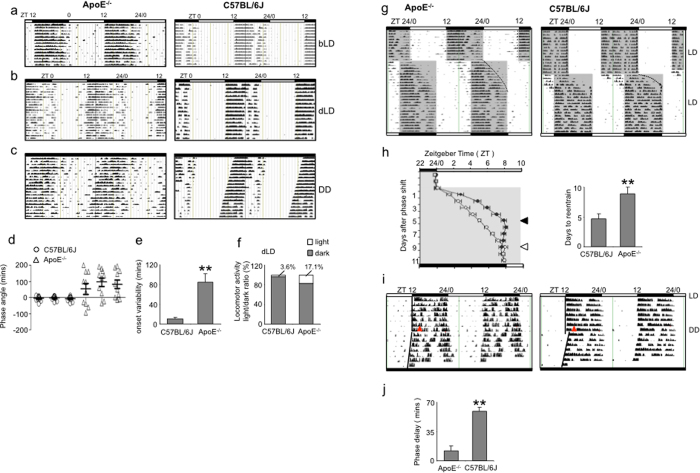
The locomotor rhythmic activities of ApoE^−/−^ mice. (**a–c**) Representative actographs showing wheel-running activity of ApoE^−/−^ and C57BL/6J mice under bLD, dLD and DD conditions after 2 weeks of LD entrainment. (**d**) Scatter dot plot of phase angle of entrainment to ZT12 for three individual ApoE^−/−^ and C57BL/6J mice were shown, with mean ± SE indicated for each mouse. The phase angle of entrainment was calculated from 10 consecutive days for each mouse. Note the greater variability in ApoE^−/−^ mice. (**e**) Onset variability at ZT12 in ApoE^−/−^ and C57BL/6J mice with mean ± SD indicated. Note the greater variability in ApoE^−/−^ mice (n = 6 for both ApoE^−/−^ and C57BL/6J mice, **indicates P < 0.01). (**f**) Locomotor activity light/dark ratio in ApoE^−/−^ and C57BL/6J mice with percentage (%) indicated. The running activities were calculated in both the light and dark phases, and the ratio was determined. (**g**) Representative actograph showing wheel-running activity of ApoE^−/−^ and C57BL/6J mice as adapted to a “jet-lag” phase delay schedule. The grey areas indicate the darkness. (**h**) To determine the days of re-entrainment, the difference between offset (activity stop point) at each day after the abrupt phase delay schedule and ZT24 in the original LD schedule, was plotted against the number of days after phase delay for ApoE^−/−^ and C57BL/6J mice until a stable re-entrainment was established. The days of re-entrainment were also presented in bar graphs with mean ± SD indicated (n = 6 for both ApoE^−/−^ and C57BL/6J mice, **indicates P < 0.01). (**i**) Representative actographs showing a light-pulse induced phase shift of ApoE^−/−^ and C57BL/6J mice. A 15-min light pulse (200 lux) was given at ZT14 to animals kept in LD schedule, and phase shifts were calculated in reference to ZT12. (**j**) Light-pulse induced phase shifts were presented as bar graphs with mean ± SD indicated (n = 6 for both ApoE^−/−^ and C57BL/6J mice, **indicates P < 0.01).

We first investigated the locomotor rhythm of the ApoE^−/−^ mice, and chose 6-week old mice to avoid the complication of aging. Compared with the C57BL/6J mice, ApoE^−/−^ mice showed relatively normal entrainment to bright light (bLD, 200 lux), with slightly increased activities in the light phase (Fig. 1a). While the C57BL/6J mice were well entrained to the dim light (dLD, 50 lux) schedule, ApoE^−/−^ mice were entrained poorly: their circadian locomotor rhythm was unstable and fragmented, with frequent activities during the latter period of light phase (Fig. 1b). Under the constant dark (DD) condition, ApoE^−/−^ mice showed substantial running activities at the subjective daytime when they should be at rest, instead of a clear-cut rest-type rhythm (Fig. 1c).

Statistically, we found no difference in total wheel running activity under three lighting conditions and the free running period under the DD condition between ApoE^−/−^ and C57BL/6J mice (Supplementary Fig. 1a,b). However under the dLD condition, the ApoE^−/−^ mice exhibited greater phase angle of entrainment with significant variation (ApoE^−/−^: 15.5 ± 23.39 mins versus C57BL/6J: 6.15 ± 4.56 mins), greater onset variability (ApoE^−/−^: 84.92 ± 16.7 mins versus C57BL/6J: 10.27 ± 2.90 mins) and greater activity light/dark ratio (ApoE^−/−^: 17.1% versus C57BL/6J: 3.6%) (Fig. 1d–f). We didn’t quantify and compare the phase angle of entrainment and onset variability under the bLD and DD conditions, because the bright light could potentially mask the locomotor activity and the exact position for CT12 appeared difficult to determine under the DD condition. The wheel running activity light/dark ratio was also higher in ApoE^−/−^ mice under the bLD condition (Supplementary Fig. 1c).

In addition, ApoE^−/−^ mice were slower to adapt to a “jet-lag” phase delay schedule, as evidenced by the reentrained time being almost twice as that of C57BL/6J mice (Fig. 1g,h). Because the difference in phase delay experiment could be due to the defect in light sensing or SCN central clock, we performed a light pulse-induced phase shift experiment to more directly assess the resetting ability of the SCN (Fig. 1i,j). As ApoE^−/−^ mice did not have a stable rhythm under the DD condition, we used Ashcroft type II protocol, in which ApoE^−/−^ mice were first kept in a bLD schedule for 7 days until a stable rhythm was attained^19^. The animals were then exposed to a light pulse at ZT14, and switch to the DD schedule. Phase shift was quantified and used as a measure of the SCN resetting ability to external light stimuli. While C57BL/6J mice displayed a significant phase shift (59.5 ± 4.20 mins), the resetting seemed blunted in ApoE^−/−^ mice (12.3 ± 5.91 mins). This result demonstrated that the SCN resetting ability was most likely impaired in ApoE^−/−^ mice. The total wheel running activity and the free running period were not altered after light pulse stimuli (Supplementary Fig. 1d,e).

**Figure 2:**
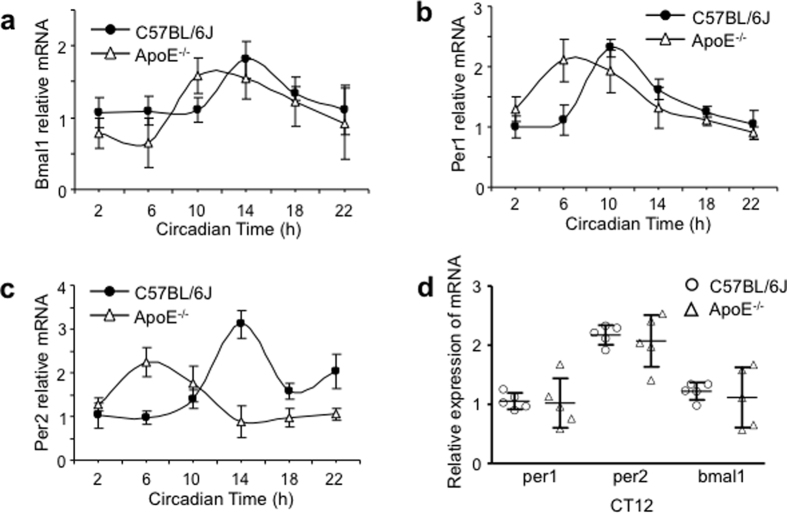
The clock gene expression in the ApoE^−/−^ and C57BL/6J SCN. (**a–c**) The circadian pattern of PER1, PER2 and BMAL1 mRNA of the SCN lysate prepared from ApoE^−/−^ and C57BL/6J mice. Note that the transcript peaks of the three genes all phase-advanced in the ApoE^−/−^ mice (n = 5 for each gene at each time point in each strain). (**d**) Scatter dot plot and mean ± SD for the transcript levels of PER1, PER2 and BMAL1 in the SCN of both strains examined at CT12. Note that the greater variability in the transcript levels in the ApoE^−/−^ mice than C57BL/6J mice (n = 5 for both ApoE^−/−^ and C57BL/6J mice).

Taken together, the behavioral studies demonstrate that the ApoE^−/−^ mice, compared with the C57BL/6J mice, had increased phase angle of entrainment with significant variation, increased onset variability, increased light/dark activity ratio, slow adaptation to phase delay schedule and poor resetting ability to light pulse. These alterations and impairments, similar to the day/night activity abnormalities in AD patients, suggest that SCN rhythmicity and/or light entrainment were impaired in ApoE^−/−^ mice. The unaltered free running period suggests that the SCN central clock was perhaps not greatly affected in ApoE^−/−^ mice at least at the age of six weeks, although other transgenic AD mouse model showed shorter period^14^. We also analyzed the circadian expression of clock genes, PER1, PER2 and BMAL1 in the C57BL/6J and ApoE^−/−^ SCN. All three genes exhibited an unstable expression pattern, with much greater variation and a noticeable phase advance in ApoE^−/−^ SCN compared with C57BL/6J SCN (Fig. 2a–d, Table 1). These changes in clock gene expression may account for the alterations observed in wheel running experiments (Fig. 1a–c).

**Figure 3:**
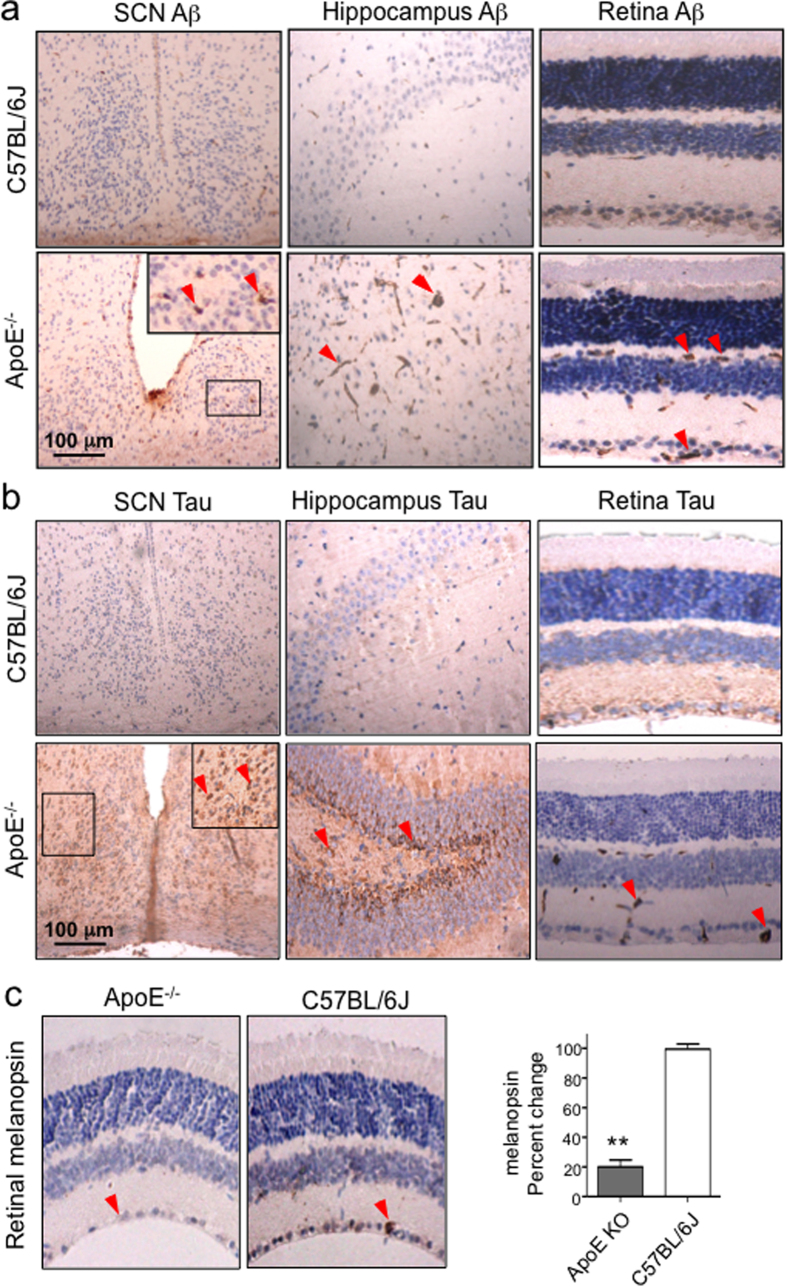
Neurodegenerative pathologies of ApoE^−/−^ mice. (**a,b**) Immunohistochemical staining of Aβ and tau in the SCN, hippocampus and retina of ApoE^−/−^ and C57BL/6J mice. The upper right large inlets in the SCN panel shows the enlarged images of the framed areas indicated by the small square. (**c**) Immunohistochemical staining of melanopsin (brown color) in the RGC layer (arrow) in the retina of the ApoE^−/−^ and C57BL/6J mice, together with image quantitation result (n = 6 for both ApoE^−/−^ and C57BL/6J mice, ** indicates P < 0.01).

Because AD is pathologically characterized by extracellular aggregates of amyloid β (Aβ) peptides and intracellular neurofibrillary tangles containing phosphorylated tau^20^, we also examined whether ApoE deficiency resulted in Aβ and tau depositions in the SCN, retina and hippocampus, despite the young age of the mice. Using immunohistochemistry, we detected significant Aβ and tau staining in the SCN, hippocampus and retina in ApoE^−/−^ but not C57BL/6J mice (Fig. 3a,b, Supplementary Fig. 2), providing proof that ApoE^−/−^ mice developed neurodegeneration at a young age and thus represented an adequate model for AD.

We next examined the expression of retinal melanopsin in ApoE^−/−^ mice. Immunohistochemistry showed that the melanopsin-expressing RGCs were sparsely located in the ganglion cell layer (Fig. 3c), consistent with previously reported expression pattern^21^. We found that the melanopsin staining was significantly lower in ApoE^−/−^ mice than the C57BL/6J mice, indicating the loss of melanopsin-expressing ganglion cells. The loss of melanopsin-expressing ganglion cells, which has been reported in AD patients^22^, confirmed that light sensing/entrainment was reduced in ApoE^−/−^ mice.

Melatonin, a neurohormone secreted mainly by the pineal gland at night, feedbacks to SCN to affect circadian rhythm and initiate sleep^23^. Melatonin has been used to treat behavioral symptoms including sundowning in AD, and reductions in melatonin production and melatonin receptor sensitivity have been reported in AD patients and elderly^24^. Surprisingly, we found no difference in the serum level of melatonin between ApoE^−/−^ and C57BL/6J mice or between ZT6 and ZT18 within a strain (Supplementary Fig. 3).

**Figure 4:**
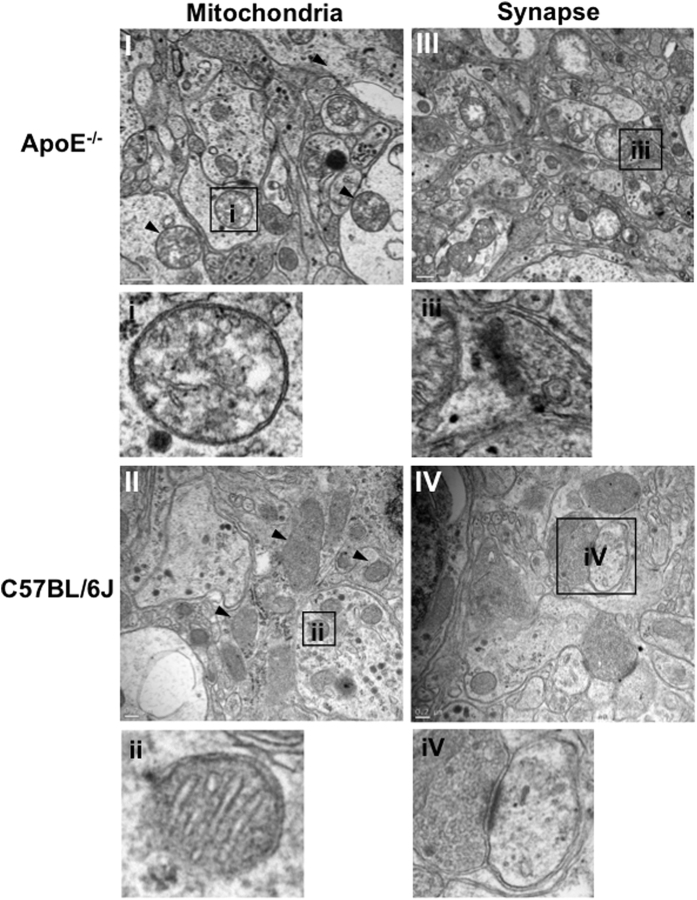
Mitochondrial and synaptic deterioration in ApoE^−/−^ SCN. Representative transmission electron microscopic images of ApoE^−/−^ and C57BL/6J SCN. Note the mitochondrial swelling and vacuolization, cristae shortening, fracturing, misaligning and disappearance (*black arrow heads*) in ApoE^−/−^ SCN, compared with the normal mitochondria of C57BL/6J SCN with the intact and well-aligned cristae (**I,II**). Also shown are the reduced synaptic structures and pre-synaptic vesicles in ApoE^−/−^ SCN, compared with the C57BL/6J SCN (**III,IV**). The small panels (**i–iv**) show the enlarged images of the framed areas indicated by the small squares in each large panel (**I–IV**).

We then used transmission electron microscopy to examine the ultrastructural changes in ApoE^−/−^ SCN (Fig. 4). Extensive mitochondrial swelling and vacuolization, cristae shortening, fracturing, misaligning and disappearance (*black arrow heads*) were seen in ApoE^−/−^ SCN, while the normal mitochondria in C57BL/6J SCN was characterized by intact and well-aligned cristae. We also found reduced pre-synaptic vesicles and synaptic structures in ApoE^−/−^ SCN. These data suggest that mitochondrial and synaptic decline, together with amyloid deposition, contributed to the SCN degeneration in ApoE^−/−^ mice.

**Figure 5:**
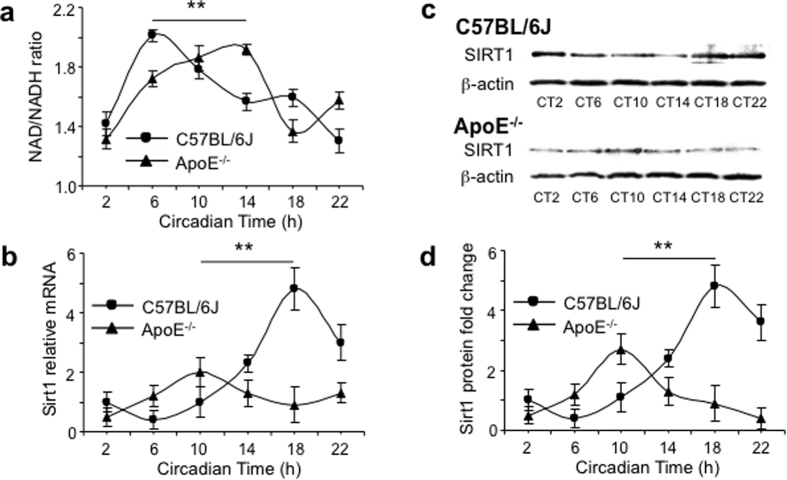
The altered SIRT1-mediated energy metabolism in ApoE^−/−^ SCN. (**a**) The circadian pattern of NAD^+^/NADH ratio of the SCN lysates prepared from the ApoE^−/−^ and C57BL/6J mice. (**b**) The circadian pattern of SIRT1 mRNA of the SCN lysate prepared from the ApoE^−/−^ and C57BL/6J mice. (**c**) The representative Western blots for SIRT1 protein of the SCN lysate prepared from the ApoE^−/−^ and C57BL/6J mice, together with the quantitative scanning densitometry result (**d**) (n = 6 for each gene at each time for both ApoE^−/−^ and C57BL/6J mice, **indicates P < 0.01). β-actin was used as a loading control. Cropped images were used and the full-length blots were presented in Supplementary Fig. 4.

SIRT1, a NAD^+^-dependent deacetylase of the mammalian sirtuin family, is essential in energy metabolism and calorie restriction^25^. SIRT1 is also known to regulate mitochondrial function and biogenesis through SIRT3, and modulate circadian rhythmicity by activating the transcription of BMAL1 and CLOCK^25^. Recently, SIRT1 was found significant in the aging of the central nervous system and pathogenesis of several neurodegenerative diseases including AD^26^,^27^. We found that the ratio of NAD^+^/NADH, two important redox co-factors, exhibited distinct circadian rhythms in ApoE^−/−^ and C57BL/6J SCN under the DD condition: 1) the peak NAD^+^/NADH ratio was at CT14 in ApoE^−/−^ SCN versus CT6 in C57BL/6J SCN, with high levels located between the CT10–CT14 (the subjective late daytime and early nighttime) and CT5–CT9 (around the subjective noon), respectively; 2) the peak value of NAD^+^/NADH ratio was lower in ApoE^−/−^ SCN (Fig. 5a). SIRT1 mRNA and protein also exhibited an evident circadian rhythm in C57BL/6J SCN (Fig. 5b,c, Supplementary Fig. 4). In ApoE^−/−^ SCN, the SIRT1 peak expression, however, was reduced and showed an 8-hour phase advance (Fig. 5b–d, Supplementary Fig. 4).

The above data demonstrate a decreased and disordered SIRT1-mediated metabolism in the mitochondria of ApoE^−/−^ SCN, leading to a shortage of energy supply. The total cholesterol (TC) level was markedly increased in ApoE^−/−^ mice, while the total triglyceride (TG) and glucose levels were less affected (Table 2)^28^. Glucose, despite being the preferred energy source, could not be efficiently utilized by the brains of ApoE^−/−^ mice and AD patients, because glucose update and metabolism were compromised in these neurons^29^,^30^. Indeed, oral supplementation with 1% glucose failed to reduce the arrhythmic locomotor activities in the light phase in ApoE^−/−^ mice under the DD condition (Supplementary Fig. 5). Multi-nutrient diets containing defined lipids have been demonstrated to improve the motor and cognitive function and reduce the Aβ deposition in ApoE^−/−^ mice and AD patients^31^. We therefore suspected that supplementation with fat, another major energy source, might more effectively improve the SCN function and restore the normal locomotor rhythmicity.

**Figure 6:**
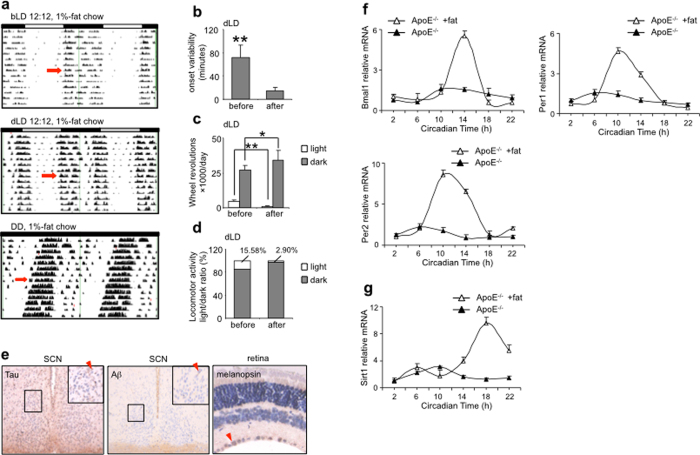
Oral supplementation with 1% fat reversed abnormal locomotor rhythmicity of ApoE^−/−^ mice. (**a**) Representative actographs showing the wheel-running activities of ApoE^−/−^ mice fed with regular chow supplemented with 1% fat for 7 days under the bLD, dLD or DD conditions (the start of the treatment indicated by solid arrows), with the quantitation results of onset variability (**b**) the running activity in the light and dark phases (**c**) and locomotor activity light/dark ratio (**d**) under the dLD condition (n = 6 for both ApoE^−/−^ and C57BL/6J mice, **indicates P < 0.01, *indicates P < 0.05). (**e**) Immunohistochemistry shows reduced the Aβ and tau staining in ApoE^−/−^ SCN and elevated melanopsin staining in the retina of ApoE^−/−^ mice fed with regular chow supplemented with 1% fat. (**f**) The circadian expression of PER1, PER2 and BMAL1 in the SCN of fat-fed ApoE^−/−^ mice. Note that the phase advance in the circadian expression of all three genes was corrected. (**g**) The circadian expression of SIRT1 mRNA expression in ApoE^−/−^ SCN. Note that the 8-hour phase advance was also reversed after fat supplementation (n = 6 for each gene at each time for both ApoE^−/−^ and C57BL/6J mice).

Oral supplementation with 1% fat for 7 days reduced the onset variability, the arrhythmic running activity in the light phase and light/dark locomotor activity ratio in ApoE^−/−^ mice under the dLD condition (Fig. 6a–d), to the extent comparable with the C57BL/6J mice fed with regular chow (Fig. 1b,d–f). Under this treatment, the running activity in the dark phase was also appreciably increased (Fig. 6c). However, no statistical difference was seen in the total running activity and free running period under the DD condition (Supplementary Fig. 6a). Fat supplementation also significantly reduced the Aβ and tau deposition in ApoE^−/−^ SCN, and improved the melanopsin expression in the retina (Fig. 6e), to the extent comparable with the C57BL/6J mice fed with regular chow (Fig. 3a–c).

We also analyzed the circadian expression of PER1, PER2 and BMAL1 in the SCN of fat-fed ApoE^−/−^ mice (Fig. 6f). The phase advance in the circadian expression of all three genes was corrected: the peak expression point located back to CT10 or CT14 (as was the case for C57BL/6J mice), and the peak expression levels of all three genes significantly increased. The 8-hour phase advance the SIRT1 mRNA expression was also reversed in ApoE^−/−^ SCN after fat supplementation (Fig. 6g). These results together demonstrate that fat supplementation effectively mitigated the degeneration in the SCN and retina in ApoE^−/−^ mice, and improved the SCN locomotor function and retinal light sensing. Fat supplementation also increased the serum TC but not TG level in ApoE^−/−^ mice, while wheel running activities decreased both TG and TC levels (Table 2).

**Figure 7:**
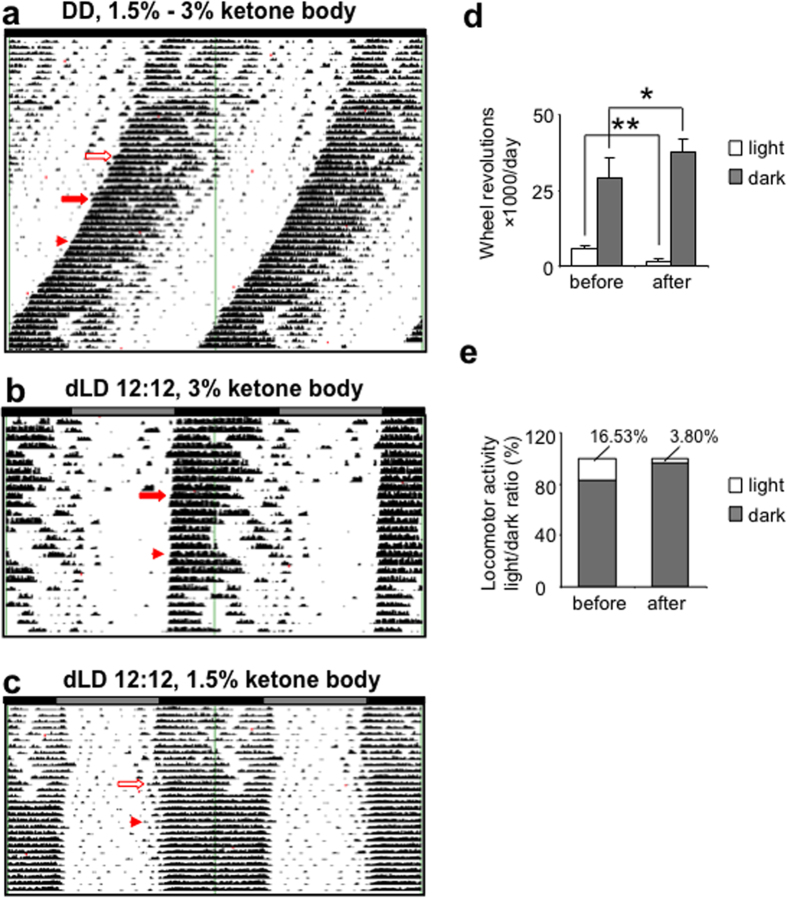
Oral supplementation with KB reversed abnormal locomotor rhythmicity of ApoE^−/−^ mice. (**a**) Representative actograph showing the wheel-running activities under the DD condition of ApoE^−/−^ mice fed with regular chow, then switched to regular chow supplemented with 1.5% followed by 3% KB each for 7 days and finally switched back to regular chow (the start of the treatment indicated by open and solid arrows for 1.5% and 3% KB respectively, and the end of the treatment indicated by arrowhead). (**b**) Representative actograph showing the wheel-running activities under the dLD condition of ApoE^−/−^ mice fed with regular chow, then switched to regular chow supplemented with 3% KB for 7 days, and finally switched back to regular chow (the start of the treatment indicated by solid arrow and the end of the treatment indicated by arrowhead). (**c**) Representative actograph showing the wheel-running activities under the dLD condition of ApoE^−/−^ mice fed with regular chow, then switched to regular chow supplemented with 1.5% KB for 7 days, and finally switched back to regular chow (the start of the treatment indicated by open arrow and the end of the treatment indicated by arrowhead). Note that 1.5% KB supplementation partially reversed, while 3% KB supplementation completely reversed the free-running arrhythmic running, and this effect lasted for 18 days after KB withdrawal. (**d,e)** Locomotor activity in the light and dark phases and light/dark ratio under the dLD condition of ApoE^−/−^ mice fed with regular chow supplemented with 3% KB (n = 6 for both ApoE^−/−^ and C57BL/6J mice, ** indicates P < 0.01, *indicates P < 0.05).

Extrahepatic tissues and especially the brain are known to utilize ketone bodies (KB), an energy source synthesized in the liver, in lieu of glucose. Ketogenic diet and ketone ester supplement have been demonstrated to improve the motor and cognitive function and reduce the Aβ deposition in ApoE^−/−^ mice and AD patients^32^. We therefore also tested whether the supply of KB reversed the CRDs in ApoE^−/−^ mice. Supplementation with KBs restored the normal locomotor rhythmicity of ApoE^−/−^ mice in a dose-dependent manner: 3% KB totally reduced the arrhythmic running in the light phase and locomotor activity light/dark ratio under the DD and dLD conditions, and the effect lasted at least 14 days after the KB withdrawal (Fig. 7a,b,d,e); 1.5% KB only partially reversed the fragmented activity-onsets (Fig. 7a,c). Similarly, the total running activity in the dark phase was also appreciably increased under the dLD condition by KB supplementation (Fig. 7d). Under these treatments, no statistical difference was observed in the total wheel running activity or the free running period under the DD condition (Supplementary Fig. 6b).

**Figure 8:**
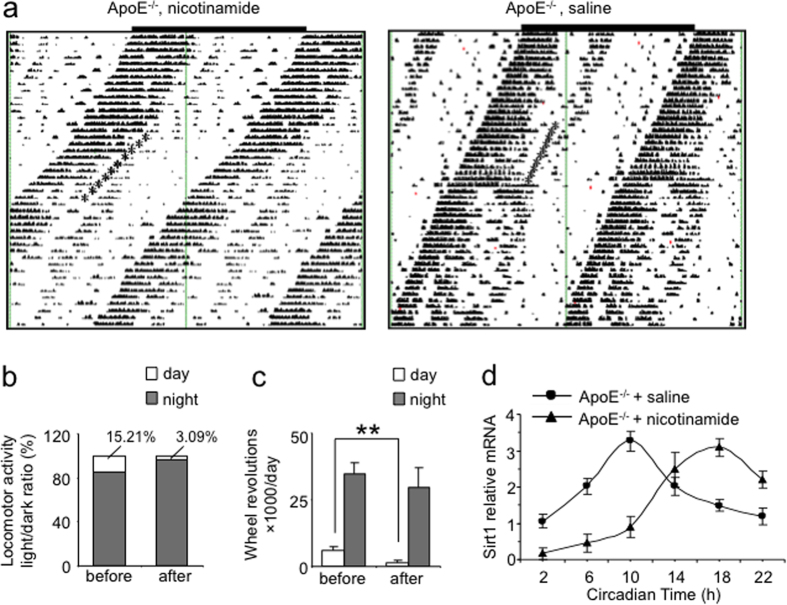
Intraperitoneal administration of nicotinamide partially reversed abnormal locomotor rhythmicity of ApoE^−/−^ mice. (**a)** Representative actograph showing the wheel-running activities of ApoE^−/−^ mice intraperitoneally injected with nicotinamide or saline at the CT22 in each day consecutively for 10 days under the DD condition (the administration at each day indicated by *). Note that the fragmented activity-onsets became well aligned during administration and lasted for 3 days after nicotinamide withdrawal. (**b,c**) Locomotor activity in the subjective day and night phases and day/night activity ratio under the DD condition of ApoE^−/−^ mice injected with nicotinamide. (**d**) The circadian expression of SIRT1 mRNA expression in ApoE^−/−^ SCN after nicotinamide administration. Note that the 8-hour phase advance was reversed (n = 5 at each time point for both ApoE^−/−^ and C57BL/6J mice, **indicates P < 0.01).

We also tested whether raising the NAD^+^ level by nicotinamide to compensate the reduced SIRT1 expression might improve the energy metabolism in ApoE^−/−^ mice and reverse the arrhythmic free-running cycles under the DD condition. Nicotinamide was intraperitoneally administered for 10 days. The fragmented activity-onsets became well aligned during administration and lasted for 3 days after nicotinamide withdrawal (Fig. 8a). The treatment partially reduced the arrhythmic running in the subjective day phase and light/dark locomotor activity ratio under the DD condition (Fig. 8b,c). Nicotinamide also reversed the phase advance of SIRT1 expression in ApoE^−/−^ SCN (Fig. 8d). Under this treatment, no statistical difference was observed in the total wheel running activity or the free running period (Supplementary Fig. 6c).

## Discussion

This study focused on whether ApoE deficiency leads to CRDs, by what mechanisms the CRDs occur, and by what means the CRDs can be rescued, aiming to identify novel curative strategies to treat the sleep disorders and CRDs in AD patients. Several important conclusions can be drawn from this study. First, ApoE deficiency leads to poor entrainment to dim light, arrhythmic free-running rhythms under the dLD and DD conditions, slow adaptation to phase delay schedule and impaired SCN resetting ability to light pulse. The circadian expression of PER1, PER2 and BMAL1 also showed phase advance, with unstable expression pattern and greater variation in ApoE^−/−^ SCN. Whether these altered circadian expressions alone result in the behavioral activity changes requires further experimental confirmation.

Complex “phosphorelay” signaling pathways were activated to enable the SCN entrained to the light signal from the retinohypothalamic tract, and these include multiple kinases, e.g. PKA, PKG, CaMK, and MAPK^33^. These pathways converge on the cAMP response element binding protein (CREB), and phosphorylated CREB binds to cAMP response elements (CRE) in the promoters of light-regulated genes, including Per1 and Per2, to activate or repress their transcription. In our studies, we didn’t find free running period significantly altered, which seemed to suggest that the SCN central clock was not greatly affected at least in 6-week old ApoE^−/−^ mice. Whether the entrainment signaling pathways were altered in ApoE^−/−^ SCN and the altered signaling pathway alone resulted in changes in locomotor rhythms requires further characterization.

A recent study showed that the locomotor rhythm and daily living activity were disrupted in transgenic mice expressing the human ApoE ε4 allele^34^. This is consistent with the notion of ApoE ε4 as a causative factor for AD, and supports our findings that ApoE deficiency leads to SCN degeneration and malfunction. In fact, mice expressing the human ApoE ε4 allele were also prone to develop sleep disruption and intermittent hypoxia^35^. These findings, together with our results, strongly suggest that abnormal ApoE expressions seen in the familial and sporadic AD result in CRDs.

Secondly, we found that the reduced expression of melanopsin in the RGCs and ultrastructural deterioration in the SCN, especially the mitochondria and synapse, were two major neuropathic mechanisms accounting for the decreased light sensing/entrainment and SCN dysfunction in ApoE^−/−^ mice. Notably, significant Aβ and tau deposition was found in the retina, SCN and hippocampus in young aged ApoE^−/−^ mice, providing proof that ApoE^−/−^ mice are prone to develop neurodegeneration.

We found no difference in the serum level of melatonin, suggesting that melatonin, which is downstream of SCN, was not involved in the CRD in ApoE^−/−^ mice. This seems to be in contrast with the reported role of melatonin in AD pathogenesis. In fact, the circadian pattern of pineal secretion of melatonin in some inbred rodent strains, including C57BL/6 mice, has been debatable. Only a tiny, short-term peak of pineal melatonin in the middle of the dark phase was observed in the C57BL/6 mice^36^. Recently, one study demonstrated that hydroxyindole O-methyltransferase, the last-step enzyme for melatonin synthesis, was not expressed in C57BL/6 strain, due to mutations^37^. Consequently, melatonin level is significantly lower in C57BL/6 mice, compared with other mouse strains. We suspect that any rhythmic change of serum melatonin (which may include extrapineal sources) may have escaped our detection, as it may be too small. Taken together, we propose that melatonin most likely does not play a significant role in the development of CRDs in ApoE^−/−^ mouse model as opposed to human.

Third, we found that the NAD^+^/NADH ratio was lower in ApoE^−/−^ SCN and exhibited a phase shift. The SIRT1 expression similarly decreased and showed an 8-hour phase advance in ApoE^−/−^ SCN. PER1, PER2 and BMAL1 also showed phase advance, with unstable expression pattern and much greater variation, in ApoE^−/−^ SCN. These data, together with the deterioration in mitochondrial morphology, strongly suggest that the metabolic function of mitochondria is compromised in ApoE^−/−^ SCN, possibly due to decreased SIRT1 expression, creating an energy shortage. Metabolic disorders including dyslipidaemia and diabetes are epidemiologically linked to AD^38^. High-fat diets are known to alter both the central and peripheral clocks, including the locomotor activity^11^,^39^. Hypothetically, the elevated cholesterol levels in ApoE^−/−^ mice could reset the SCN central clock. The resulting CRDs could trigger and influence the process of neurodegeneration.

The transcription of nicotinamide phosphoribosyltransferase (NAMPT), a rate-limiting enzyme for NAD^+^ biosynthesis, is controlled by the CLOCK-BMAL1 complex^40^. NAMPT generates an oscillating NAD^+^ rhythm, which couples the mitochondrial oxidative phosphorylation with the circadian clock. NAD^+^ rhythm also regulates SIRT1 activity and clock gene expression^41^. Our data that the SIRT1-mediated energy metabolism, clock gene expression and mitochondrial morphology all similarly declined in ApoE^−/−^ SCN support such an interconnected regulatory mechanism comprising the clock circuit, mitochondria and SIRT1 in the SCN circadian biology.

Fourth, based on an “energy shortage” mechanism underlying the CRD in ApoE^−/−^ mice, we devised experiments to improve the calorie supply and compensate the reduced SIRT1 activity. Our results showed that these regimens, which are either moderate oral supplementation with common energy sources or administration of clinically proven vitamin, reversed the arrhythmic locomotor activity in ApoE^−/−^ mice. Such rescue experiments are significant in the sense that they not only restore the running rhythm, but also reduce the morphological neurodegeneration and reverse arrhythmic clock gene expression in the case of fat supplementation. Future experiments are needed to establish how and in which temporal order mitochondria metabolic dysfunction, circadian arrhythmicity and neurodegeneration were improved in the SCN neurons, although we strongly suspect that these processes are closely intertwined with each other.

In this study, we have only examined the effect of 1% fat but not KB or nicontinamide on reducing the Aβ and tau deposition in ApoE^−/−^ SCN and improving the melanopsin expression in the retina. Although we assume that both KB and nicontinamide would improve the energy supply to SCN, further experiments are needed to establish whether KB and nicontinamide indeed reverses the neurodegenerative morphologies in the same manner as fat supplementation. It also remains to be established whether the circadian expression of clock genes is reversed by these treatments in the SCN, although the circadian expression of SIRT1 was restored by nicotinamide. Prolonged dietary and pharmacological interventions are required to assess the long-term beneficial effects, such as complete annihilation of CRDs and complete reversal of neurodegeneration. We reason that the regimens in our study are directly applicable to future dietary or pharmacological therapies to treat the CRDs in both the familial and sporadic AD patients.

## Methods

### Animals

Six-week-old male ApoE^−/−^ mice and age-matched male C57BL/6J mice were purchased from the Laboratory Animal Center of Peking University and the Experimental Animal Center of Chinese Academy of Medical Sciences (Beijing, China). Animals were housed in a temperature-controlled room (25 ± 1 °C) equipped with the VitalView Data Acquisition System (Mini-Mitter Inc., USA), and maintained under a 12:12 h light-dark (LD 12:12) cycle (light on at 06:00, off at 18:00) for at least 2 weeks before the start of experiments. Chow and water were provided without restriction. The animal protocol followed the “Principles of Laboratory Animal Care” (NIH publication No. 86–23, revised 1985), and was approved by the Ethics Committee of Peking Union Medical College. All the animal studies complied with the ARRIVE guidelines.

All chemicals were purchased from Sigma Aldrich unless stated otherwise. Pelleted diets were used in all our experiments. For standard locomotive experiments, C57BL/6J and ApoE^−/−^ mice received normal pelleted diet. For curative experiments, neutral fat was added to regular chow at 1% (w/w), and the fat-containing pelleted diet was prepared by Experimental Animal Center of Chinese Academy of Medical Sciences (Beijing, China). ApoE^−/−^ mice were fed with the fat-chow for 7 days. Ketone ester (MCE, catalogue # HY15344, USA) was added to regular chow at 1.5% or 3% (w/w) and the mixed pelleted diet was prepared in-house. The mice were fed with the KB-containing chow for 7 days. Nicotinamide (Sigma Aldrich, catalogue # 72340, USA) was dissolved in saline and intraperitoneally injected at 400 mg/kg body weight/day at CT22 consecutively for 10 days. Equivalent volume of saline was injected as a vehicle control.

### Recording of wheel-running activities and animal grouping

ApoE^−/−^ and C57BL/6J mice were managed to perform voluntary wheel-running activities inside the facility as described previously^42^. Data analyses were performed using the ActiView Biological Rhythm Analysis software. After a minimum of 2-week entrainment to LD 12:12 schedule (light intensity 200 lux), half of the mice were kept in the LD 12:12 circumstance, which were further divided to two groups exposed to bright light (200 lux):dark (bLD 12:12) or dim light (50 lux):dark (dLD 12:12) condition, respectively. A *zeitgeber time (ZT)* system was used in the LD 12:12 schedule, light on at 06:00 (ZT0), and light off at 18:00 (ZT12). Another half of mice were placed into a constant dark (DD) circumstance for 3 weeks after a period of acclimation to the LD 12:12 schedule, and a *circadian time (CT)* system was used. The onset, start point of wheel-running activities, was set as CT12, i.e., the beginning of subjective night.

After wheel running experiments, total running activities (wheel revolutions), the free-running periods, the running activities in the light and dark phases and locomotor activity light/dark ratio were determined using the ActiView Biological Rhythm Analysis software, and statistically quantified. For ApoE^−/−^ mice injected with nicotinamide, the mice were kept under the DD condition. Total wheel running activities in the subjective day and subjective night phases were quantified, and day/night (light/dark under bLD and dLD conditions) ratios were deduced. For the determination of phase angle of entrainment, we calculated the difference between onset (the start point of wheel-running activities) and ZT12 (the light off point) for each mouse every day for a period of 10 consecutive days. Means and standard errors were calculated after the statistical analysis of phase angle of entrainment for each mouse. A large standard error indicates significant onset variability. Onset variability was defined as the absolute difference between onset activity and ZT12. Means and standard errors were calculated after the statistical analysis.

Some animals from both strains underwent a “jet-lag” light schedule after the above LD 12:12 entrainment. “Jet lag” was created by an abrupt transition from the LD 12:12 schedule to the LD 20:4 schedule, i.e., an 8-hour prolongation of the light phase, which corresponded to an 8-hour light phase delay or an “east-to-west” jet-lag, followed by the LD 12:12 schedule with a new start point of light phase. To determine the days of re-entrainment, the difference between offset (activity stop point) at each day after the abrupt phase delay schedule and ZT24 in the original LD schedule, was plotted against the number of days after phase delay for ApoE^−/−^ and C57BL/6J mice until a stable re-entrainment was established. When a stable re-entrainment was ultimately established, the difference between offset at that day and ZT24 in the original bLD schedule was defined as days of re-entrainment.

To analyze the SCN resetting ability, animals were kept in a bLD schedule for 7 days until a stable rhythm had been recorded. The animals were then exposed to 200 lux of light for 15 min at ZT14, and switch to the DD schedule. Phase shift was defined as a difference between onset of activity (the start of the post-stimuli line) and ZT12 (the pre-stimuli line extrapolated to the first activity onset after the light pulse), and was calculated using eye-fit lines drawn by at least 3 independent observers through consecutive onsets of activity.

### Tissue sampling

Animals were anaesthetized with sodium pentobarbital (15 mg/50 g body weight) after wheel-running experiments. Then the animals were decapitated, retina and brain tissues (SCN and hippocampus) were harvested as described previously^42^, and frozen with liquid nitrogen for RT-qPCR and Western blotting, or fixed with 4% formalin for immunohistochemistry. For measuring the NAD^+^/NADH levels, the circadian expression of clock genes (BMAL1, PER1, PER2) and SIRT1, SCN tissues were obtained at CT2, CT6, CT10, CT14, CT18 and CT22 under a dim red light (<4 lux).

### Immunohistochemistry (IHC)

After the wheel-running experiments, eyes and brains were harvested and fixed with 4% formalin. Then, retina, SCN and hippocampus were carefully dissected, routinely developed and embedded in paraffin wax. Five μm sections were cut and mounted onto slides. Sections were deparaffinized, rehydrated and then reacted with a 3% hydrogen peroxide/methanol solution to inactivate endogenous peroxidase, followed by a final 5 min wash with phosphate buffer solution (PBS). A two-step immunohistochemical staining protocol was used. The sections were incubated with the primary antibodies against melanopsin (Pierce, catalogue # PA-781, USA), Aβ (Abcam, catalogue # ab-11132, USA) and tau (CST, catalogue # 4019, USA), respectively for 2 h, washed with PBS, and then incubated with the biotinylated secondary antibody (Jackson ImmunoResearch Laboratories Inc., USA). The immunoreactive proteins were visualized with a liquid DAB substrate-chromogen system (ZSGB-BIO, China). The sections were finally rinsed with water, dehydrated through a graded alcohol series, and mounted. Parallel control experiments were conducted without adding the primary antibody. To determine the level of melanopsin in the retina, Aβ and tau in retina, SCN and hippocampus, images were captured with a camera. Density analyses of immunolabeled signals were conducted using the BI-2000 Medical Image Analysis System (Chengdu Taimeng Software, China) as previously described^42^. Fold change relative to control (C57BL/6J mice) was plotted as the expression level of melanopsin, Aβ and tau for ApoE^−/−^ mice. For the immunostaining quantitation, two consecutive slides from three separate animals were selected and examined to obtain the mean and SD value.

### Western blotting

SCN samples or retina tissues were lysed in radioactive immunoprecipitation (RIPA) buffer and total proteins were extracted. To obtain enough retina proteins, retinas of three animals were pooled. The protein concentration was determined using a bicinchoninic acid kit following the manufacture’s protocol. Total protein (50 μg) was applied to each lane on 10% SDS-polyacrylamide gels. After electrophoretic transfer, the nitrocellulose membranes were incubated with the primary antibodies (anti-SIRT1: Santa Cruz Biotechnology, catalogue # sc-15404, USA). β-actin was used as a loading control. The labeled proteins were detected using an enhanced chemiluminescence detection kit (Amersham ECL, USA). Then the images were captured and quantitation was performed using ImageQuant LAS 4000 mini (GE Healthcare, USA). The average value of three independent experiments was used for the results for each time point.

### Transmission electron microscopy (TEM)

TEM was performed to examine the degenerative changes of SCN neurons. Mice were anesthetized with sodium pentobarbital (15 mg/50 g body weight) and fixed by transcardial perfusion with 300 ml of 1% paraformaldehyde and 1% glutaraldehyde in 0.1 M phosphate buffer (pH 7.3). Coronal sections (1 μm) of the SCN tissues were cut with a vibratome and postfixed by incubation for 1 hour in 1% osmium tetroxide. These sections were then dehydraded by passage through a series of concentrations of ethanol and embedded in Araldite. Ultrathin sections (60 nm thick) were cut from the blocks (1 mm^3^) prepared from the vibratome sections. SCN ultrastructures, especially the mitochondria and synapse, were examined, and images were taken under a transmission electron microscope (JEOL-1011, Japan) at 80 kV with a GATAN digital camera (Gatan, USA).

### Real-time quantitative PCR (RT-qPCR)

After the wheel-running experiments, animals were decapitated under dim red light (<4 lux), and SCN tissues were immediately harvested and stored in liquid nitrogen. SCN was homogenized in Trizol (Invitrogen, USA) and total RNAs were extracted. Single-strand cDNA was prepared from 2 μg total RNA by reverse transcription. RT-qPCR was performed on StepOnePlus^TM^ Real-time PCR system (Applied Biosystems, CA, USA), with SYBR Green PCR Master Mix and primers specific for mouse BMAL1, PER1, PER2, SIRT1 and β-actin. Briefly, both β-actin (used as a control) and the target gene from the same sample were amplified in duplicate separate tubes for each assay. The mRNA levels of each gene were calculated using the relative standard curve and normalized against the corresponding β-actin level, and then expressed as relative change over control. The primers were designed using the Primer Express V1.5 software (Applied Biosystems, CA, USA) as follows: BMAL1: forward, 5′-TGGCCGCTGTAGACACTACATT; reverse, 5′-CTCTATCCAGTAAGCTTCACAGACTGTAA; PER1: forward, 5′-TCGAAACCAGGACACCTTCTCT; reverse, 5′-GGGCACCCCGAAACACA; PER2: forward, 5′-ATGCTCGCCATCCACAAGA; reverse, 5′-GCGGAATCGAA TGGGAGAAT; SIRT1: forward, 5′-CAGGTTGCAGGAATCCAAA; reverse, 5′-CAAATCAGGCAAGCTGCTGT; β-actin: forward, 5′-TCAAGATCATTGCTCCTCCTGAG; reverse, 5′-CTGCTTGCTGATCCACATCTG. The results represent the average of three independent experiments for each time point.

### Measurement of NAD^+^/NADH

NAD^+^/NADH ratio was used to estimate the energy metabolism status of SCN. NAD^+^ and NADH nucleotides were measured by an enzymatic NADH recycling assay, using a NAD^+^/NADH quantification kit (Abcam, USA) following the manufacturer’s instruction. Each SCN tissue (20 mg) was washed with cold PBS and homogenized using a Dounce homogenizer with 400 μl of NADH/NAD extraction buffer in a microcentrifuge tube. The homogenate was centrifuged at 14,000 rpm for 5 min. The supernatant containing the NADH/NAD was transferred to a new tube and kept on ice. Fifty μl of the extracted samples were transferred to a 96-well plate, and the total NAD (total NAD and NADH) and NADH were determined. The intracellular concentrations of NAD, NADH and their ratios were calculated from a NADH standard curve.

### Enzyme linked immunosorbent assay (ELISA)

Serum melatonin levels were measured with a mouse melatonin ELISA kit. Briefly, blood samples of about 500 μl were taken from the eyes of mice at ZT6 (noon) and ZT18 (midnight) under anesthesia. Blood samples were kept static in a tube for 1 hour and then centrifuged at 2500 rpm for 15 min to obtain the serum for ELISA. A standard curve of melatonin levels was prepared, and ELISA was performed following the manufacturer’s instruction. Optical absorbance were measured at 450 nm and corrected by 570 nm. Data were analyzed using SoftMax Pro4.8 software.

### Statistical analysis

Data were presented as mean ± standard deviation (SD). Student’s *t* tests or ANOVA were performed for comparison of group differences wherever necessary. The criterion for statistical significance was *P* < 0.05.

## Additional Information

**How to cite this article**: Zhou, L. *et al*. Degeneration and energy shortage in the suprachiasmatic nucleus underlies the circadian rhythm disturbance in ApoE^−/−^ mice: implications for Alzheimer’s disease. *Sci. Rep.* **6**, 36335; doi: 10.1038/srep36335 (2016).

**Publisher’s note:** Springer Nature remains neutral with regard to jurisdictional claims in published maps and institutional affiliations.

## Supplementary Material

Supplementary Information

## Figures and Tables

**Table 1:** The transcript levels of PER1, PER2 and BMAL1 in the SCN examined at CT12.

	PER1		PER2		BMAL1	
	C57BL/6J	ApoE^−/−^	C57BL/6J	ApoE^−/−^	C57BL/6J	ApoE^−/−^
Mean	1.054	1.022	2.174	2.072	1.222	1.116
SD	0.1392	0.4182	0.1656	0.4388	0.1470	0.5093

Mean and SD were shown for each group (n = 6).

**Table 2:** Serum lipid levels of mice under different dietary and locomotor activity conditions.

Animal and treatment	n	TG (mmol/L)	TC (mmol/L)
C57BL/6J, regular chow, WR (−)	6	1.73 ± 0.18	3.98 ± 0.47
ApoE^−/−^, regular chow, WR (+)	6	0.91 ± 0.11**	6.48 ± 0.63**
ApoE^−/−^, fat chow, WR (+)	6	1.89 ± 0.16^ΔΔ^	6.94 ± 0.67
ApoE^−/−^, fat chow, WR (−)	6	2.27 ± 0.24^#^	12.63 ± 1.65^##^

TG, total triglyceride; TC, total cholesterol; WR (+), with wheel running; WR (−), without wheel running. **P < 0.01 *vs.* the respective TG or TC level of C57BL/6J mice with regular chow but without wheel running. ^ΔΔ^*P* < 0.01 *vs*. the TG level of ApoE^−/−^ mice with 1%-fat chow and wheel running. ^#^*P* < 0.05 *vs.* the TG level of ApoE^−/−^ mice with 1%-fat chow and wheel running. ^##^*P* < 0.01 *vs.* the TC level of ApoE^−/−^ mice with 1%-fat chow and wheel running. Mean and SD were shown for each group (n = 6).
