# Supplementary material for: Degeneration and energy shortage in the suprachiasmatic nucleus underlies the circadian rhythm disturbance in ApoE−/− mice: implications for Alzheimer’s disease
Source: Sci Rep. 2016 Nov 8;6:36335. doi: 10.1038/srep36335 (PMC5099891; doi:10.1038/srep36335)
Supplement: Supplementary Information [file srep36335-s1.pdf]

Degeneration and energy shortage in the suprachiasmatic nucleus underlies the circadian rhythm disturbance in ApoE$^{-/-}$ mice, implications for Alzheimer's disease

Lan Zhou, Qian Gao, Meng Nie, Jing-Li Gu, Wei Hao, Lin Wang and Ji-Min Cao

Figure S1

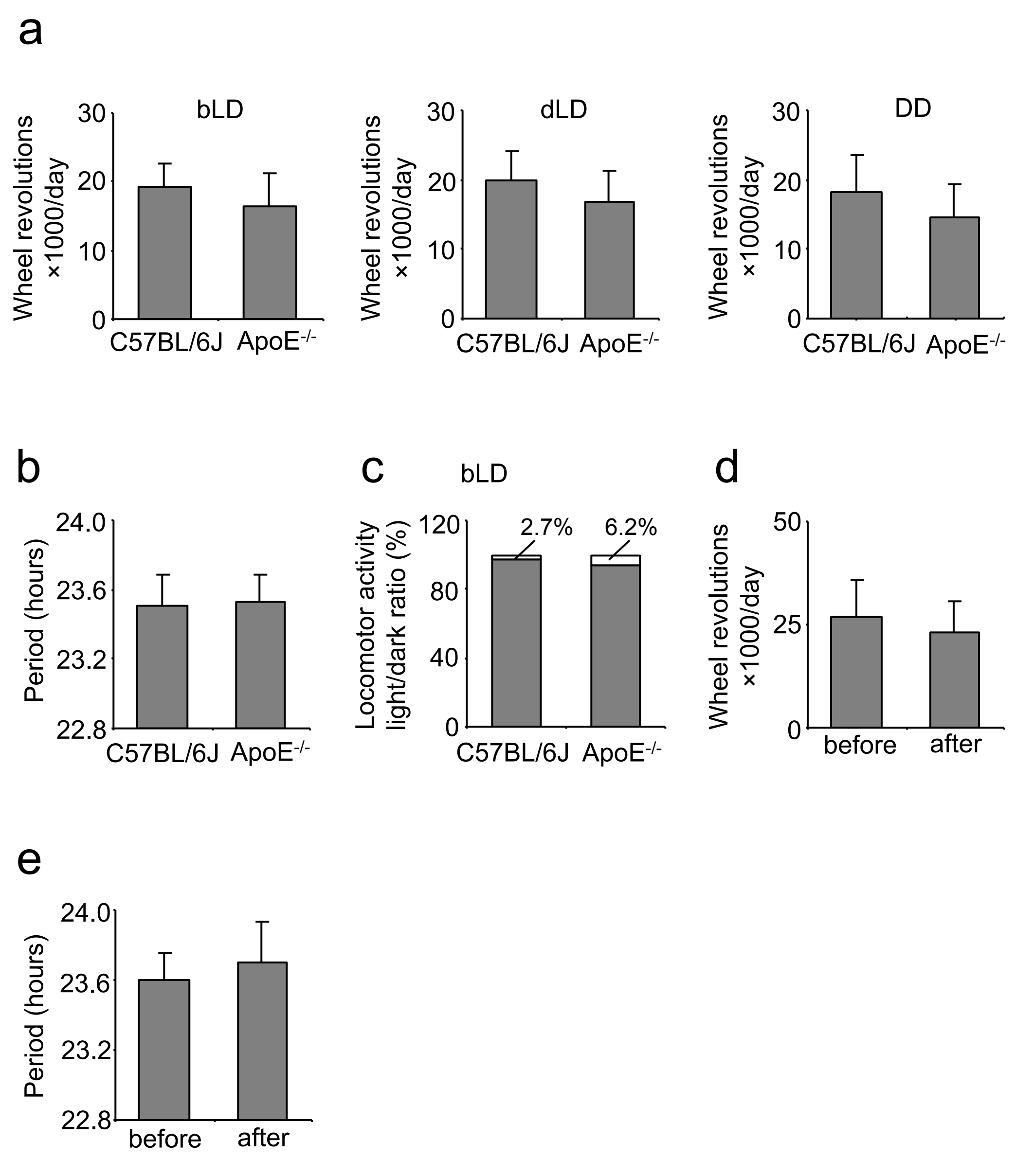

Fig. S1 The free running period and total wheel running activity of ApoE$^{-/-}$ and C57BL/6J mice. **(a)** Total wheel running activity of ApoE$^{-/-}$ and C57BL/6J mice under three lighting conditions. **(b)** The free running period under the DD condition of ApoE$^{-/-}$ and C57BL/6J mice. **(c)** The wheel running activity light/dark ratio in ApoE$^{-/-}$ versus C57BL/6J mice under the bLD condition. **(d,e)** The total wheel running activity and the free running period were not altered after light pulse. In all the experiments, n = 6 for both ApoE$^{-/-}$ and C57BL/6J mice.

Degeneration and energy shortage in the suprachiasmatic nucleus underlies the circadian rhythm disturbance in ApoE$^{-/-}$ mice, implications for Alzheimer's disease
Lan Zhou, Qian Gao, Meng Nie, Jing-Li Gu, Wei Hao, Lin Wang and Ji-Min Cao
Figure S2

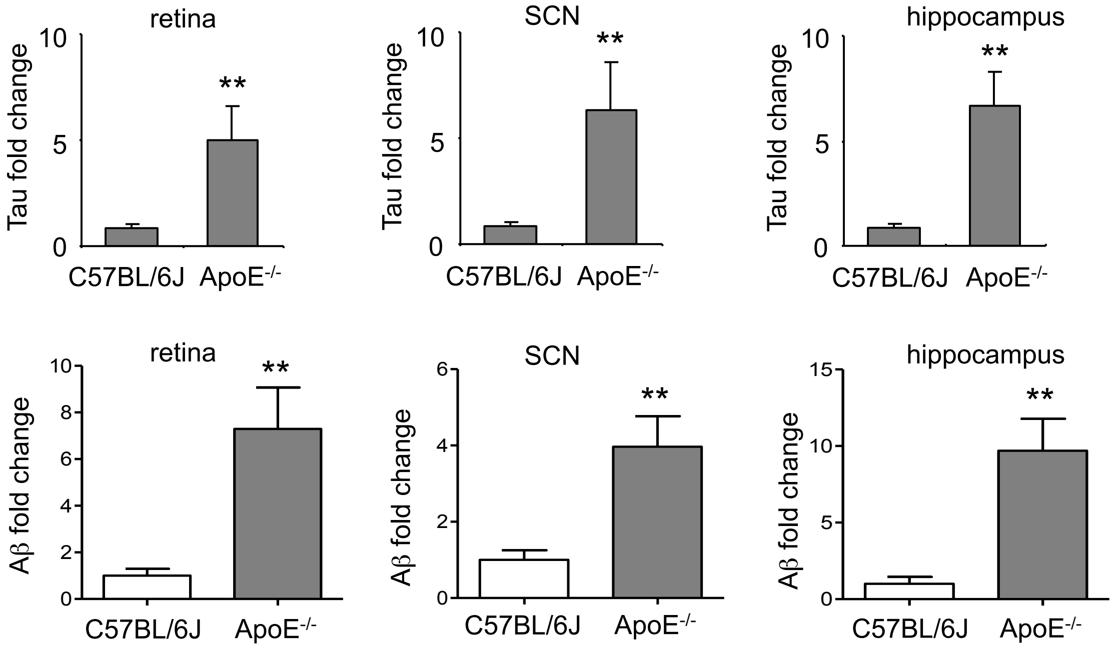

Fig. S2 Quantitation of immunohistochemistry staining of Aβ and tau in the SCN, hippocampus and retina in ApoE$^{-/-}$ and C57BL/6J mice (n = 6, ** indicates $P < 0.01$)

Degeneration and energy shortage in the suprachiasmatic nucleus underlies the circadian rhythm disturbance in ApoE$^{-/-}$ mice, implications for Alzheimer's disease

Lan Zhou, **Qian Gao**, **Meng Nie**, **Jing-Li Gu**, **Wei Hao**, **Lin Wang and Ji-Min Cao**

Figure S3

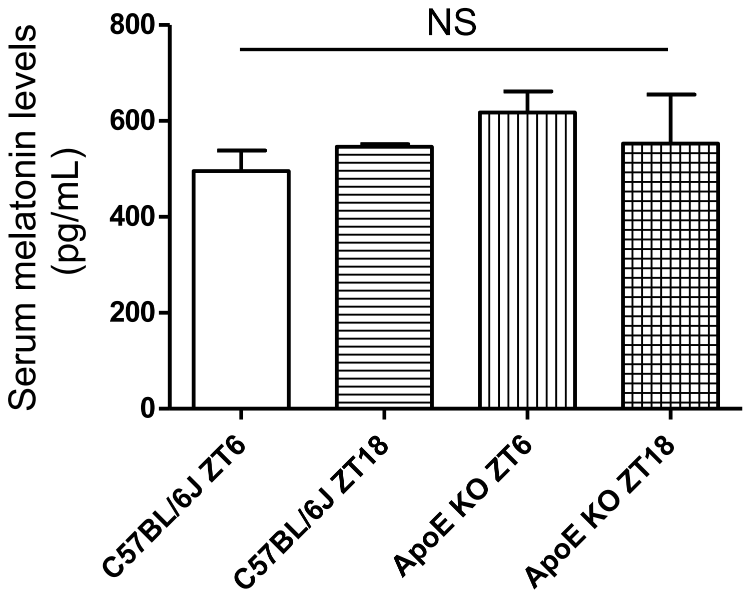

Fig. S3 Serum melatonin levels of ApoE$^{-/-}$ and C57BL/6J mice.
Bar graph shows the mean ± SD of serum melatonin levels of the two strains measured by ELISA at ZT6 and ZT18 (n = 6). Student t-test was performed to confirm that there was no statistical significance between the two strains or between the two ZT points within a strain. NS: not statistically significant.

Degeneration and energy shortage in the suprachiasmatic nucleus underlies the circadian rhythm disturbance in ApoE$^{-/-}$ mice, implications for Alzheimer's disease

Lan Zhou, Qian Gao, Meng Nie, Jing-Li Gu, Wei Hao, Lin Wang and Ji-Min Cao

Figure S4

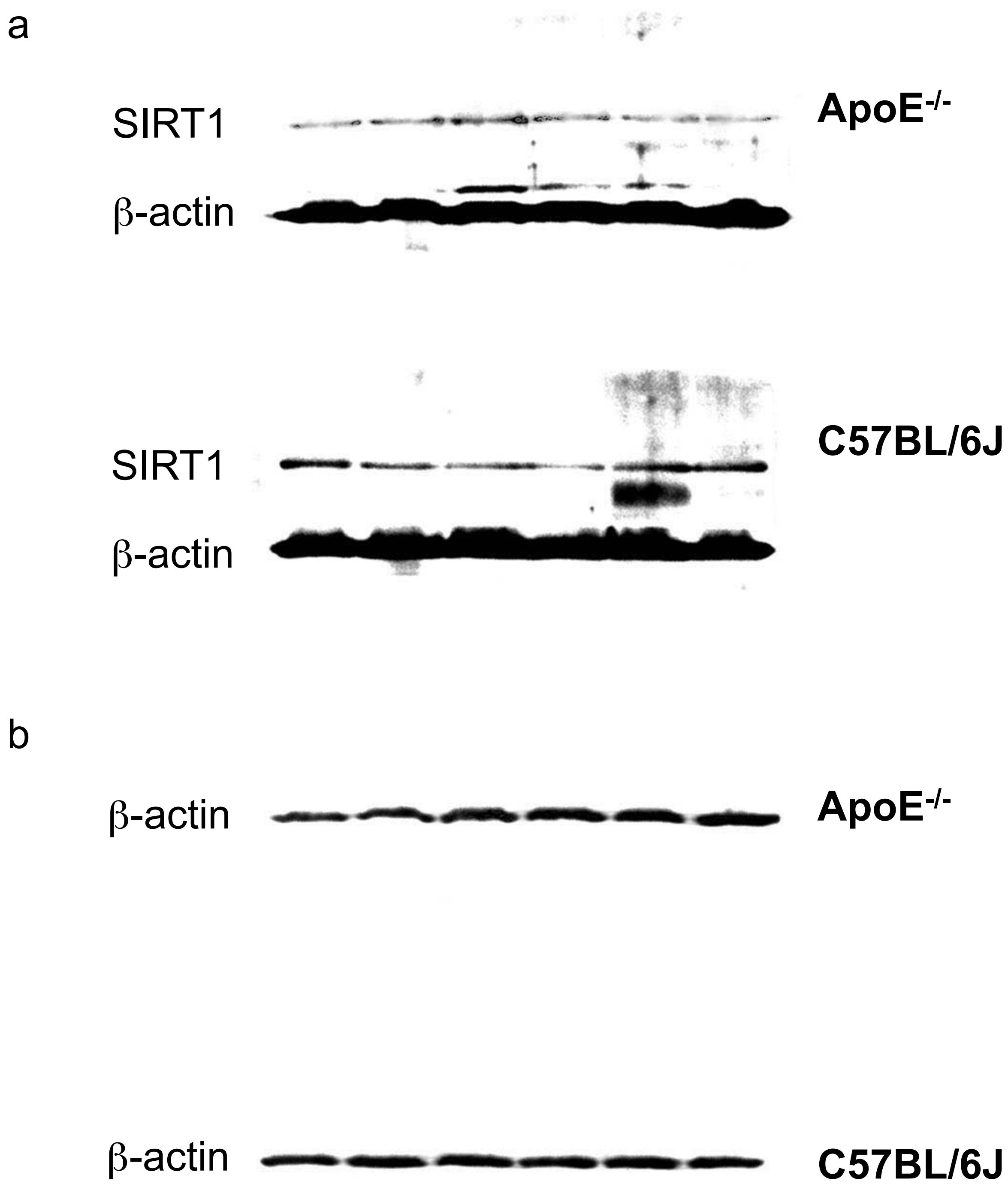

Fig. S4 The full-length blots for SIRT1 and β-actin from ApoE$^{-/-}$ and C57BL/6J SCN. The images of SIRT1 and β-actin after ECL detection were captured using ImageQuant LAS 4000 mini and a series of exposures were taken. A long (a) and short (b) exposure are shown. Some bands appear none specific. Only the bands with the relevant molecule size were cropped and presented in Fig. 5.

Degeneration and energy shortage in the suprachiasmatic nucleus underlies the circadian rhythm disturbance in ApoE$^{-/-}$ mice, implications for Alzheimer's disease

Lan Zhou, Qian Gao, Meng Nie, Jing-Li Gu, Wei Hao, Lin Wang and Ji-Min Cao

Figure S5

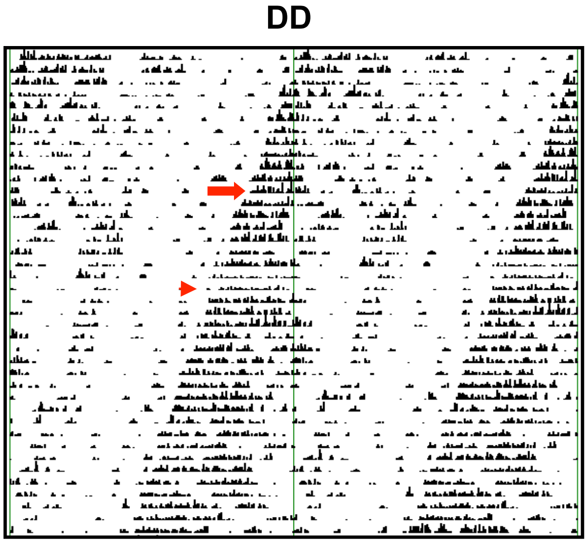

Fig. S5 Glucose supplementation was unable to reverse the CRDs in ApoE$^{-/-}$ mice under the DD condition.

Representative actograph showing the wheel-running activity of ApoE$^{-/-}$ mice fed with regular chow, then switched to regular chow containing 1% of glucose (w/w) for 7 days and finally switched back to regular chow (the start of the treatment indicated by solid arrow and the end of the treatment indicated by arrowhead).

Degeneration and energy shortage in the suprachiasmatic nucleus underlies the circadian rhythm disturbance in ApoE$^{-/-}$ mice, implications for Alzheimer's disease

Lan Zhou, Qian Gao, Meng Nie, Jing-Li Gu, Wei Hao, Lin Wang and Ji-Min Cao

Figure S6

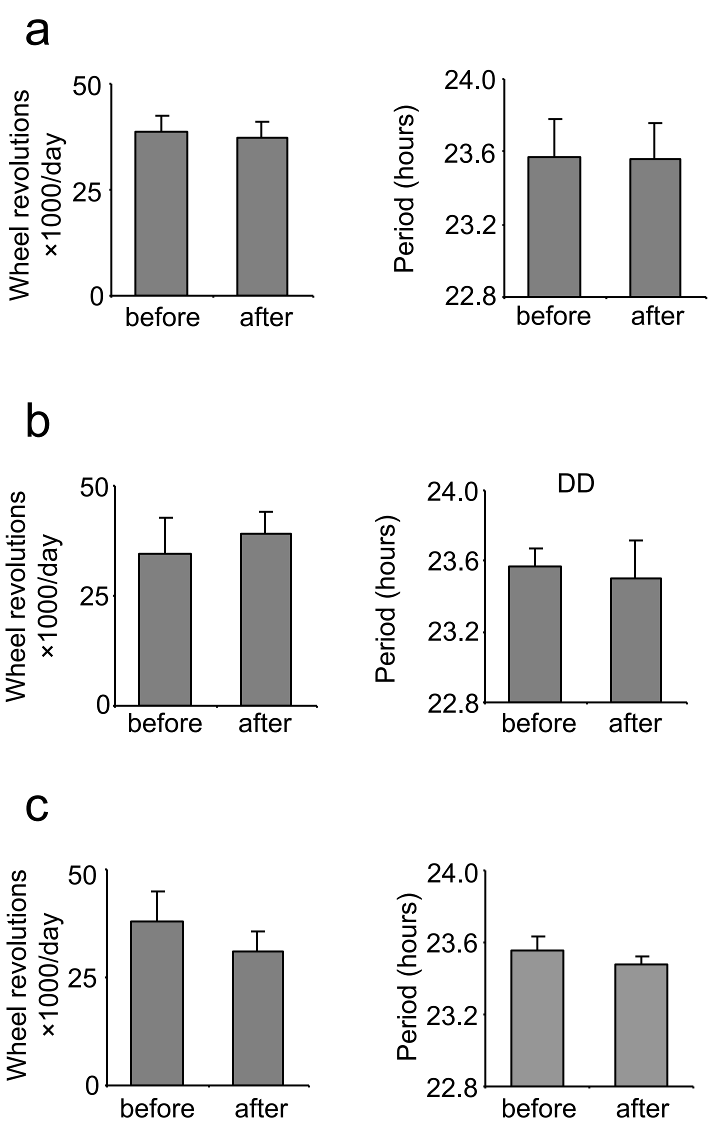

Fig. S6 Total running activities and free running period of ApoE$^{-/-}$ mice after fat or KB supplementation or intraperitoneal administration of nicotinamide.

Oral supplementation with fat **(a)** or KB **(b)** or intraperitoneal administration of nicotinamide **(c)** did not alter total wheel running activities and free running period of ApoE$^{-/-}$ mice (n = 6 for both ApoE$^{-/-}$ and C57BL/6J mice).
